# Low-dose multi-walled carbon nanotubes are non-inflammatory alone but amplify lipopolysaccharide-induced cytokine responses in A549 lung epithelial spheroids

**DOI:** 10.3389/fimmu.2026.1794151

**Published:** 2026-07-15

**Authors:** Jian Liu, Li Zeng, Wondwossen Abate, Simon K. Jackson

**Affiliations:** 1 School of Biochemistry and Biomedical Sciences, Faculty of Health and Life Sciences, University of Bristol, Bristol, United Kingdom; 2 Shanxi Academy of Medical Sciences, Taiyuan, China; 3 Clinical and Biomedical Sciences, University of Exeter Medical School, Exeter, United Kingdom; 4 School of Biomedical Science, Faculty of Health, University of Plymouth, Plymouth, United Kingdom; 5 Molendotech Ltd, Brixham Laboratory, Brixham, Devon, United Kingdom

**Keywords:** carbon nanotubes, epithelial spheroid, inflammatory cytokine, lipopolysaccharide, synergistic effect

## Abstract

**Background:**

Airborne pollutants comprise biological agents such as lipopolysaccharide (LPS) and engineered nanomaterials, including carbon nanotubes (CNTs), which are increasingly prevalent in industrial and consumer applications. Although the pulmonary toxicity of high-dose CNT exposure is established, the inflammatory consequences of low-dose, non-cytotoxic CNT exposure, particularly in combination with other aerosols, remain poorly understood.

**Methods:**

We investigated cytotoxicity and proinflammatory responses to multi-walled CNTs in three-dimensional A549 human epithelial spheroids, compared with A549 monolayers and MM6 monocytes. Cells were exposed to two CNTs differing in diameter (CNT1: 7–15 nm; CNT2: 110–170 nm), alone or combined with LPS. Cell viability was assessed by WST-1 assay and F-actin junction staining, while IL-8 and IL-6 production was quantified by ELISA and qPCR.

**Results:**

A549 spheroids were more susceptible to CNT-induced cytotoxicity than monolayers, with CNT1 inducing greater cytotoxicity than CNT2. At non-cytotoxic concentrations, CNTs alone did not elicit cytokine release; however, co-exposure with LPS significantly enhanced cytokine secretion in spheroids, particularly in the presence of CNT2. This synergistic inflammatory response was not observed in MM6 monocytes.

**Conclusion:**

Our study demonstrates that non-cytotoxic CNTs can potentiate LPS-driven inflammation in A549 epithelial spheroids, suggesting potential respiratory risks from combined exposure to environmental endotoxins and nanomaterials, even at low doses.

## Introduction

Inhaled lipopolysaccharide (LPS) is a potent activator of the innate immune system and has been implicated in airway inflammation, disease exacerbation in asthma and chronic obstructive pulmonary disease (COPD), and the pathogenesis of acute lung injury (ALI), acute respiratory distress syndrome (ARDS), and pulmonary fibrosis (1–3). Although early-life exposure to low levels of LPS may contribute to immune maturation and reduced risk of atopic disease, the relationship between LPS exposure and allergic or inflammatory airway disorders remains complex, and the increasing prevalence of these disorders is likely influenced by multiple environmental and genetic factors (4–6).

Growing evidence indicates that co-existing environmental nano-pollutants act as important immune modulators (7, 8). Among these, human exposure to carbon nanotubes (CNTs), nanoscale carbon allotropes, is rising rapidly due to their expanding use in industrial and consumer applications. Inhalation of airborne CNTs is of particular concern, as the airway epithelium serves as the primary site of exposure and a critical regulator of pulmonary immune responses (7). The potential cytotoxicity of CNTs to the human airway depends on factors such as diameter and surface chemistry (9). Compared with single-walled CNTs (SWCNTs), multi-walled CNTs (MWCNTs) are more widely produced and used in industry (10). MWCNTs are generally considered more inflammogenic, partly due to their multi-layered composition and greater effective surface area, which can enhance biological reactivity and proinflammatory signaling (11).

Most *in vitro* studies of CNT-induced toxicity or inflammation use medium to high concentrations (20-100 µg/ml) (8), whereas lower concentrations (≤ 1 µg/ml) are generally considered non-toxic and non-inflammatory (12). At higher doses, MWCNTs (20-100 µg/ml), alone or with LPS, induce pro-fibrogenic activation of alveolar epithelial cells (8). Importantly, LPS can also modulate nanoparticle-cell interactions, providing bacterial protection while increasing susceptibility to particle-induced inflammatory injury (8, 13).

*In vivo*, pre-exposure to MWCNTs amplifies LPS-induced lung inflammation and fibrosis (14). An intratracheal mouse exposure of 4 mg/kg CNTs roughly approximates an estimated *in vitro* concentration of 100 µg/ml, based on assumptions of lung volume and body weight (14). However, such comparisons should be interpreted cautiously due to substantial differences between *in vivo* pulmonary and *in vitro* submerged exposure conditions, including particle deposition, clearance, and cellular dosimetry (15). Moreover, occupational CNT exposure is typically measured as airborne elemental carbon or particle concentration (µg/m³), and direct correspondence to experimental concentrations remains challenging (15). Nevertheless, while such high experimental doses may be more relevant to certain occupational exposures (e.g., manufacturing workers, firefighters) or extreme traffic pollution scenarios, they do not represent typical daily environmental exposures (16). Meanwhile, little is known about whether sub-microgram, non-toxic CNT doses modulate epithelial inflammatory responses under co-exposure with LPS. Addressing this gap is critical because environmental and occupational exposures frequently involve complex mixtures of particulates and biological agents rather than single insults.

In parallel, ethical and regulatory pressures to reduce animal experimentation have increased reliance on advanced *in vitro* models. However, most CNT studies still employ two-dimensional (2D) monolayer cultures, which inadequately reflect the structural complexity, cell-cell interactions, and barrier properties of the lung epithelium. In contrast, three-dimensional (3D) culture systems more closely mimic *in vivo* tissue architecture, exhibiting enhanced intracellular communication and physiological barrier function, as shown by us and others (17–19). Consequently, 3D epithelial models are well suited for investigating respiratory responses to pollutants and pathogens and for improving preclinical assessments (20, 21).

In this study, we evaluated the cytotoxicity of two MWCNTs with distinct diameters and examined whether non-toxic, non-inflammatory concentrations of MWCNTs modulate LPS-induced proinflammatory cytokine production. Using an established 3D spheroid model of A549 human lung epithelial cells, we compared responses with those observed in conventional 2D monolayers and MM6 monocytes, highlighting dose-dependent and context-specific effects of CNT-LPS co-exposure.

## Methods

### Cells

The human lung epithelial cell line A549 (American Type Culture Collection, Teddington, UK) and the human monocytic cell line MM6 (German Collection of Microorganisms and Cell Cultures, Braunschweig, Germany) were cultured in RPMI1640 (Sigma-Aldrich, Gillingham, UK) supplemented with 10% fetal bovine serum, 1% nonessential amino acids, 200 nM L-glutamine, 1 mM sodium pyruvate, 100 U/ml penicillin and 100 µg/ml streptomycin (Thermo Fisher Scientific, Paisley, UK). A549 monolayers were routinely sub-cultured using 0.05% trypsin/0.02% ethylenediaminetetraacetic acid (Sigma-Aldrich) at a split ratio of 1:5. Sub-cultured A549 cells were allowed to recover for at least two days before forming spheroid cultures. MM6 cell suspensions were sub-cultured every four days at a split ratio of 1: 5.

### Gyrotatory spheroids

A549 spheroids were generated using a gyratory-mediated method described by us (19, 22). Briefly, confluent A549 monolayers were detached using 0.05% trypsin/0.02% EDTA to yield single-cell suspensions. Aliquots of 3 ml containing 1 × 10^6^ cells were transferred to 6-well plates and placed on a gyratory shaker (New Brunswick, St. Albans, UK). Plates were rotated at 83 rpm during the first 24 h to promote cell aggregation, followed by rotation at 77 rpm for the remainder of the culture period. During the initial phase, small and irregular cell aggregates progressively joined to form larger, more compact structures. After 3 days of culture, spheroids with diameters ranging from 50-100 μm were formed (**Supplementary Figure 1A**), and their size increased progressively over time (**Supplementary Figure 1B**). The A549 spheroids displayed a compact architecture with tightly packed cells and extensive cell-cell contacts, as demonstrated by F-actin staining, which was evident throughout the 3D structure for up to 9 days in culture (**Supplementary Figure 1B**). Three-day-old spheroids were selected for treatment to minimize the risk of reduced cell viability within the spheroid, a phenomenon associated with mass transport limitations that typically occur at diameters exceeding 150-200 μm (23).

### CNT suspension preparation

Two MWCNTs (Sigma-Aldrich), including the thinner CNT1 (7–15 nm in diameter and 0.5-10 μm in length, #412988) and the thicker CNT2 (110–170 nm in diameter and 5-9 μm in length, #659258), were used in this study. Immediately before cell treatment, CNTs were dispersed non-covalently in cell culture medium containing 1% bovine serum albumin (BSA; Sigma-Aldrich) to preserve their native surface chemistry and intrinsic physicochemical properties (24). BSA acts as a dispersant, reducing CNT agglomeration (25).

Suspensions were ultrasonically dispersed in a water bath for 30 min (60 W, with 30-s cooling intervals per minute on ice) as described previously (26–29), to achieve homogeneous dispersion. Samples were visually inspected to exclude macro-aggregates and applied to spheroids under continuous gyrotatory shaking, a low-shear dynamic condition that minimizes sedimentation and secondary aggregation (30). Additionally, MWCNTs, compared with SWCNTs, have greater rigidity and larger diameters, reducing tightly bundled aggregates and enabling more stable dispersions (31).

Endotoxin levels in the supernatants of the homogeneous suspensions were confirmed to be below 0.01 EU/ml using a Recombinant Factor C Assay (Lonza, Cambridge, UK). This control ensured that the observed cytokine responses were attributable to CNT/LPS co-exposure rather than unintended microbial contamination. The final CNT concentrations for cell treatment ranged 0.1-100 µg/ml.

### Cell treatment and analyses

Cells were seeded in 6-well plates (1 × 10^6^ cells/3 ml per well) and exposed for 24 h to LPS (1 µg/ml; *E. coli* 0111:B4, Sigma-Aldrich), CNT (0.1-100 µg/ml), or their combination, followed by analysis of cell viability, junctional F-actin staining, IL-8 and IL-6 immunoassay, and quantitative PCR (**Supplementary Methods**). Spheroid morphology and size uniformity were assessed by scanning electron microscopy (**Supplementary Methods**). Untreated cells under identical culture conditions served as controls for each cell or culture type.

### Statistics

Statistical analyses were conducted using Prism 9.0. Data were analyzed using one-way ANOVA followed by Bonferroni *post-hoc* comparisons for all experiments involving more than two groups and one variable. Differences at *p* < 0.05 were considered significant. Results are presented as means ± standard deviation (SD).

## Results

### CNTs elicit greater cytotoxicity in A549 spheroids than in monolayers in a diameter-dependent manner

**Figure 1:**
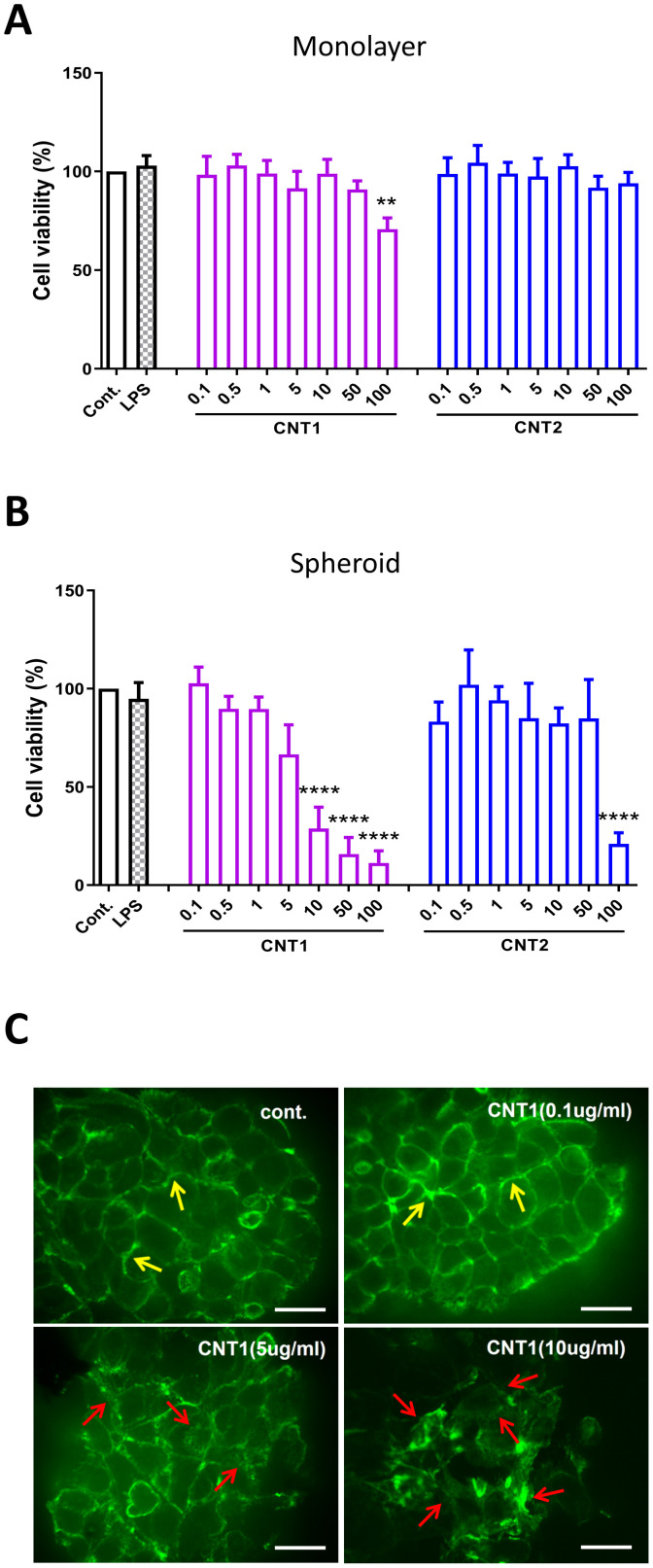
Greater cytotoxicity of CNTs in A549 spheroids than in monolayers and dose-dependent disruption of F-actin in spheroids. **(A, B)** A549 monolayers **(A)** or spheroids **(B)** were treated with LPS (1 µg/ml) or two CNTs (0.1-100 µg/ml) for 24 h. The thinner CNT1 (7–15 nm) and the thicker CNT2 (110–170 nm) were used. Cell viability was measured using WST-1 reagent. **(C)** After treatment of spheroids with CNT1 at 0.1, 5 or 10 µg/ml for 24 h, F-actin was stained with Phalloidin-Alexa Fluor 488 and observed under a confocal microscopy. Yellow and red arrows indicate normal and impaired cell-cell contacts, respectively. Scale bar: 10 µm. Data were analyzed by one-way ANOVA followed by Bonferroni *post-hoc* tests. n=3-6. **p < 0.01, ****p < 0.0001 *vs* control **(A, B)**.

Confluent A549 monolayers matched for passage number were used for comparison with spheroids. LPS (1 µg/ml) did not affect cell viability in either monolayer or spheroid cultures (**Figures 1A, B**). Interestingly, cells in monolayers were resistant to CNT exposure. Significant cytotoxicity was observed only for the thinner CNT1 (7–15 nm) at the highest concentration tested (100 µg/ml), whereas the thicker CNT2 (110–170 nm) showed no cytotoxic effects (**Figure 1A**). In pronounced contrast, cytotoxicity to spheroids was observed at 5 µg/ml CNT1 or 100 µg/ml CNT2 (**Figure 1B**).

Consistent with this increased sensitivity, cortical F-actin at cell-cell junctions, which remained intact in untreated spheroids, became progressively disrupted with increasing CNT1 concentrations (**Figure 1C**). Notably, CNT1 at 5 µg/ml disrupted F-actin organization without significantly reducing cell viability (**Figure 1B**). Based on these findings, non-cytotoxic CNT concentrations (0.1-1 µg/ml) were selected for subsequent experiments.

#### Non-cytotoxic CNT doses do not elicit IL-8 or IL-6 production but amplify LPS-induced cytokine responses in A549 spheroids

Consistent with our previous work, LPS (1 µg/ml) did not induce cytotoxicity but significantly increased secretion of IL-8 and IL-6 in A549 spheroids. We next assessed whether non-cytotoxic CNTs induce cytokine production or modulate LPS-induced inflammatory responses.

**Figure 2:**
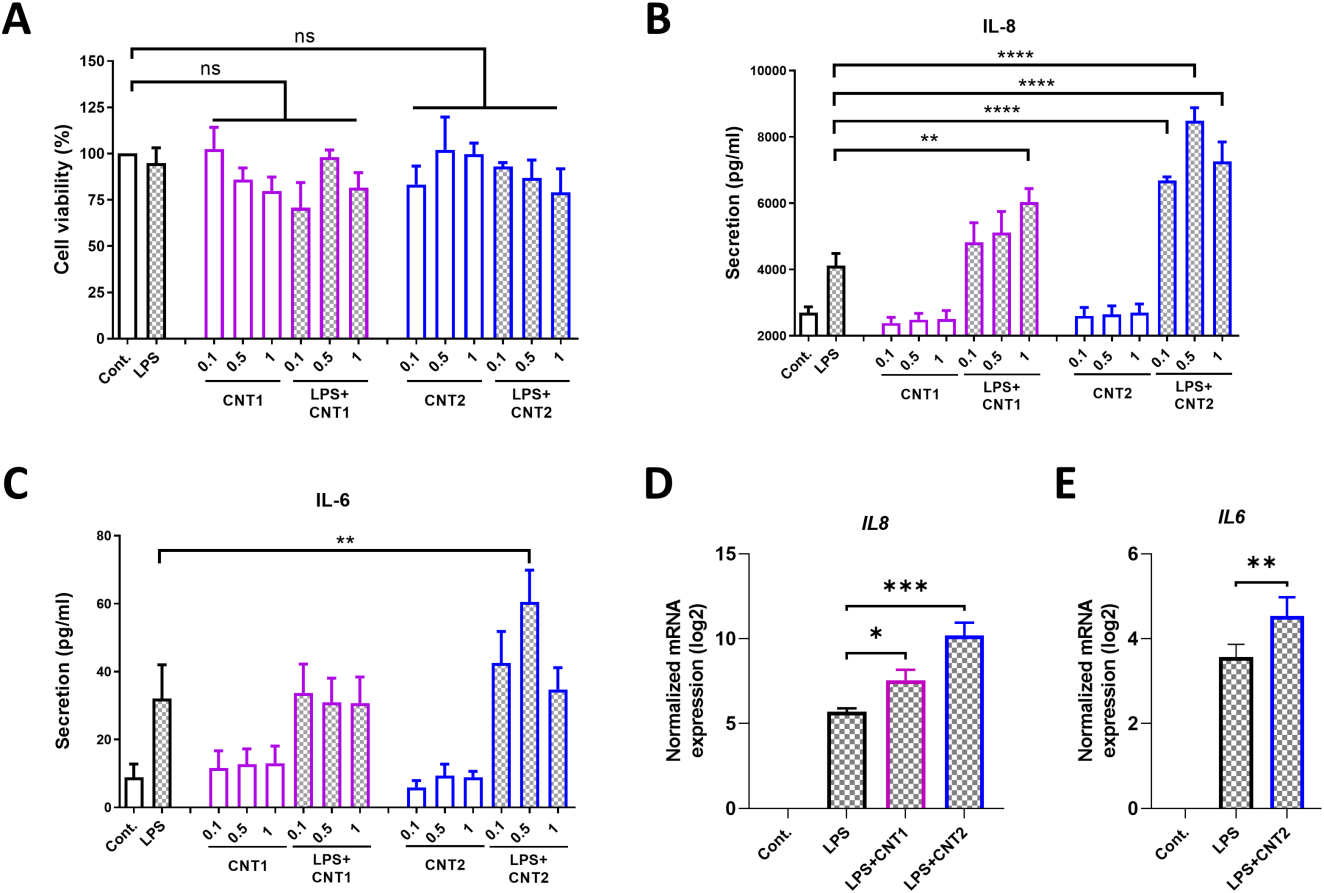
Enhanced cytokine response to LPS by non-cytotoxic, non-inflammatory CNT doses in A549 spheroids. **(A)** A549 spheroids were treated with LPS (1 µg/ml), CNT1 (0.1-1 µg/ml), CNT2 (0.1-1 µg/ml), or a combination of LPS and one of the CNTs for 24 h. Cell viability was assessed by WST-1 assay to exclude cell death-caused passive cytokine release. **(B, C)** After treatment, concentrations of IL-8 **(B)** and IL-6 **(C)** in supernatant of spheroids were measured by ELISA. **(D, E)** Spheroids were collected after 3 h of treatment for analysis of *IL8* **(D)** and *IL6* **(E)** mRNA levels by qPCR. Data were analyzed by one-way ANOVA with Bonferroni *post-hoc* tests. n=3-6. *p < 0.05, **p < 0.01, ***p < 0.001, ****p < 0.0001 **(B–E)**, ns, non-significant **(A)**.

Co-treatment with CNTs (0.1-1 µg/ml) and LPS did not affect cell viability (**Figure 2A**). Neither CNT1 nor CNT2 alone induced IL-8 or IL-6 secretion (**Figures 2B, C**), but both significantly enhanced cytokine production induced by LPS compared with LPS alone (**Figures 2A–C**). While CNT1 enhanced LPS-induced IL-8-release at 1 µg/ml, CNT2 elicited this amplification at all tested concentrations (0.1-1 µg/ml) (**Figure 2B**). For IL-6, CNT1 had no effect on LPS-induced secretion, whereas CNT2 intensified it at 0.5 µg/ml (**Figure 2C**).

This enhancement was corroborated at the transcriptional level. After 3 h of co-treatment with LPS plus CNT1 (1 µg/ml) or CNT2 (0.5 µg/ml), which are concentrations associated with maximal cytokine secretion, *IL8* and *IL6* mRNA expression was significantly higher than with LPS alone (**Figures 2D, E**).

#### Non-cytotoxic CNT doses have no effect on basal or LPS-induced cytokine production in MM6 monocytes

**Figure 3:**
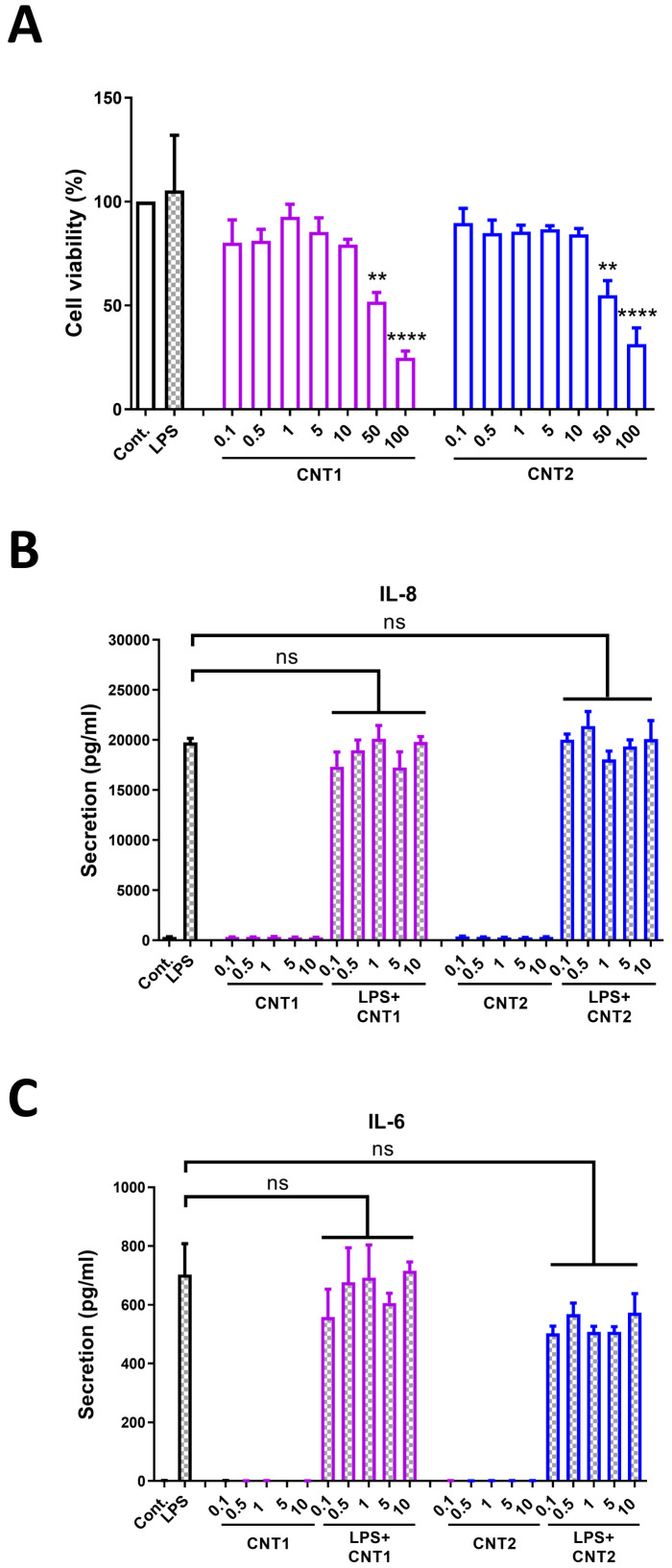
Cell viability and cytokine secretion of MM6 monocytes in response to LPS and CNT co-exposure. **(A)** MM6 cell suspensions were treated with LPS (1 µg/ml) or CNTs (0.1-200 µg/ml) for 24 h, followed by cell viability assessment using WST-1 assay. **(B, C)** Non-toxic doses of CNT1 or CNT2 (0.1-10 µg/ml) were used alone or in combination with LPS in monocyte treatment for 24 h. IL-8 **(B)** and IL-6 **(C)** concentrations in cell culture supernatants were analyzed using ELISA. Data were analysed by one-way ANOVA followed by Bonferroni *post-hoc* tests. n=3. **p < 0.01, ****p < 0.0001 *vs* control **(A)**. ns, non-significant **(B, C)**.

Monocytes are significantly influenced by various stimuli, including LPS and nanoparticles (32). To compare epithelial and monocytic responses to CNT exposure, we examined cytotoxicity and cytokine production in MM6 monocyte suspensions. LPS (1 µg/ml) did not affect monocyte viability, whereas both CNT1 and CNT2 significantly reduced viability at concentrations ≥ 50 µg/ml, independent of CNT diameter (**Figure 3A**).

As expected, LPS robustly induced IL-8 and IL-6 secretion in MM6 monocytes (**Figures 3B, C**), consistent with their high expression levels of surface receptors (33). In contrast, neither CNT1 nor CNT2 at non-cytotoxic concentrations (0.1-10 µg/ml) induced cytokine secretion or altered LPS-induced IL-8 or IL-6 production (**Figures 3B, C**).

## Discussion

This study demonstrates that sub-microgram concentrations of MWCNTs, while neither cytotoxic nor intrinsically proinflammatory, potentiate LPS-induced cytokine production in A549 lung epithelial spheroids. In contrast, despite robust IL-8 and IL-6 responses to LPS treatment in MM6 monocytic-like cells, non-cytotoxic CNT concentrations did not enhance LPS-induced cytokine production under the conditions tested.

The magnitude of CNT-LPS combined effect was dependent on CNT diameter. The thicker CNT2, despite lower cytotoxicity than the thinner CNT1, elicited greater amplification of LPS-induced IL-8 and IL-6 secretion. IL-8 and IL-6 are important mediators of airway epithelial inflammatory responses and are commonly induced following epithelial activation by microbial or environmental stimuli (19, 34–36). Previous studies reported limited LPS responsiveness and minimal CD14 expression in A549 monolayers compared with primary epithelial cells (37). However, our previous work demonstrated enhanced CD14 and MD2 expression, together with enhanced LPS-induced IL-8 and IL-6 responses, in A549 spheroids compared with monolayer cultures, suggesting that 3D culture conditions may partially improve A549 epithelial innate immune responsiveness (19), although inflammatory cytokine responses in this model may still differ from those of primary human airway/alveolar epithelium. These findings suggest that diameter-dependent physicochemical properties, such as surface characteristics, rigidity, or endotoxin adsorption, may influence epithelial immune signaling (38).

Although CNT concentrations below 10 µg/ml are often considered biologically inert, prior studies report increased IL-8 secretion and oxidative stress at 5 µg/ml (26) and mutagenic DNA damage at concentrations as low as 0.05-2 µg/ml (39). Extending these findings, our study provides the first evidence that sub-microgram MWCNTs can elicit inflammatory responses in A549 lung epithelial spheroids under co-exposure with LPS. These results highlight limitations in cytotoxicity-based safety assumptions and emphasize the importance of evaluating epithelial and monocytic inflammatory responses under co-exposure conditions. While informative, these findings were obtained using simplified *in vitro* models and warrant confirmation in more physiologically complex systems, including primary cells, co-culture, and *in vivo* models.

The heightened responsiveness of A549 spheroids supports the utility of simplified 3D epithelial models for studying epithelial inflammatory responses. Compared with monolayer cultures, 3D spheroids provide enhanced cell-cell interactions that partially improve physiological relevance under defined *in vitro* conditions (21, 40). Consistent with this, we previously demonstrated enhanced barrier integrity, adhesion molecule expression, LPS receptor expression and cytokine responses in A549 spheroids under defined *in vitro* conditions compared with monolayer cultures (19). In the present study, altered F-actin organization was observed without significant reduction in cell viability; however, epithelial barrier integrity was not directly assessed, and therefore these findings alone may not be sufficient to demonstrate epithelial barrier dysfunction.

While intracellular localization of CNTs has been reported at concentrations around 1 µg/ml (39), uptake at lower doses is poorly characterized. Limited internalization at ultra-low doses may favor extracellular or membrane-associated interactions, potentially enhancing LPS adsorption, ligand presentation, or clustering of pattern-recognition receptors including TLR4. This hypothesis may partly explain the non-linear dose response observed, with maximal cytokine amplification at intermediate CNT concentrations (**Figures 2B, C**). Such interactions could constitute a key mechanism exacerbating LPS-induced inflammatory cytokine release. Although CNT-LPS co-treatment did not significantly reduce cell viability under the tested conditions, indirect or secondary mechanisms contributing to cytokine release cannot be fully excluded and warrant further investigation.

CNT-LPS interactions may influence epithelial inflammatory responses under defined co-exposure conditions. Pre-existing microbial exposure exacerbates CNT-induced pathology in animal models, including enhanced fibrotic and inflammatory responses (41). In the present study, low-level CNT exposure, in the absence of significant cytotoxicity, amplified epithelial cytokine production in response to LPS in A549 spheroids. While awaiting confirmation in more physiologically relevant *in vitro* models (42, 43) and *in vivo* studies, such interactions may be relevant to occupational and urban environments characterized by complex co-exposures.

Several limitations should be noted. First, this study focused on two commercially available MWCNT types differing in diameters; thus, the findings cannot be generalized to CNTs with other lengths, surface chemistry, or functionalization states. Second, only LPS was evaluated as a co-exposure stimulus, and interactions with other environmentally relevant pollutants, such as particulate matter, or additional microbial components, were not assessed. Third, although the A549 spheroids provide a controlled and reproducible epithelial model, they are generated from a cancer-derived cell line and do not fully recapitulate the phenotype, polarization, multicellular organization, innate immune signaling, or cytokine responsiveness of native human airway/alveolar epithelium, and lack immune, vascular, and systemic components. Fourth, while the MM6 cells were included as a complementary monocytic-like model to assess inflammatory responses under CNT-LPS co-exposure conditions, they are transformed leukemia-derived cells and do not fully represent primary human monocytes or epithelial-immune interactions *in vivo*. Finally, the mechanisms underlying CNT-mediated amplification of LPS signaling remain unexamined and warrant further investigation into CNT-LPS interactions, TLR4 receptor clustering, and downstream MyD88- and NF-κB-dependent signaling pathways.

In conclusion, sub-microgram MWCNTs enhanced LPS-induced epithelial inflammatory responses in A549 spheroids without significantly affecting cell viability under the tested conditions. These findings, derived from a defined 3D A549 spheroid model, provide insight into epithelial responses to CNT-LPS co-exposure conditions and highlight the importance of evaluating combined nanomaterial and microbial exposures. While informative, these results warrant confirmation in more physiologically complex *in vitro* systems, including polarized and multicellular models such as air-liquid interface cultures or precision-cut lung slices (42, 43), as well as *in vivo* studies. By defining these responses in a reproducible epithelial system, this work provides a foundation for future studies investigating additional CNT types, diverse co-exposures, and the molecular mechanisms underlying CNT-LPS interactions under more physiologically relevant conditions.

## Data Availability

The raw data supporting the conclusions of this article will be made available by the authors, without undue reservation.
