# Supplementary material for: Low-dose multi-walled carbon nanotubes are non-inflammatory alone but amplify lipopolysaccharide-induced cytokine responses in A549 lung epithelial spheroids
Source: Front Immunol. 2026 Jul 15;17:1794151. doi: 10.3389/fimmu.2026.1794151 (PMC13414821; doi:10.3389/fimmu.2026.1794151)
Supplement: Supplementary file 1 [file DataSheet1.pdf]

Supplementary Methods

WST-1 assay

After treatment with LPS and/or CNTs, A549 monolayers were gently scraped, and all cells were collected by centrifugation (500 × g, 5 min, 4 ºC) and resuspended in 400 µl fresh medium. WST-1 reagent (40 µl; Roche, Mannheim, Germany) was added, and samples were incubated at 37 °C for 60 min. Following centrifugation to remove insoluble materials, 100 µl of supernatant was transferred to a 96-well plate, and absorbance was measured at 450 nm using a Multiscan RC microplate reader (Labsystems, Basingstoke, UK). Cell viability was expressed as a percentage of untreated controls for each cell type and culture condition.

F-actin staining and confocal microscopy

As previously described (17), spheroids were fixed in 90% ethanol for 20 min on ice, washed with PBS, permeabilized with 0.1% Triton X-100 for 30 min, and blocked with 1% BSA for 30 min. Junctional F-actin was stained with Phalloidin-Alexa Fluor 488 (10 U/ml; Thermo Fisher Scientific) for 20 min, and samples were mounted in VectaShield containing DAPI. Confocal images were acquired using an UltraView spinning-disk confocal microscope (Perkin Elmer, Waltham, MA) and visualized with Volocity.

Scanning electron microscopy

As previously described (14), spheroids were collected, washed with PBS, and fixed in 4% glutaraldehyde (TAAB Laboratories, Aldermaston, UK). After washing, samples were dehydrated through a graded ethanol series (20-100%) and transferred to ethanol-hexamethyldisilazane (HMDS; Sigma-Aldrich) mixture (2:1, 1:1, and 1:2). Following a final rinse in HMDS, spheroids were sputter-coated with gold and examined using an XL30 Environmental Scanning Electron Microscope (ESEM; Philips-FEI, Eindhoven, The Netherlands).

Immunoassay

After treatment with LPS, CNT, or their combination for 24 h, cell culture supernatants were collected and stored at −80°C until analysis of IL-8 and IL-6 concentrations by ELISA (R&D Systems, Abingdon, UK) according to the manufacturer’s instructions.

Quantitative PCR

Cellular RNA was isolated using TRIzol reagent (Thermo Fisher Scientific) and digested with RQ1 RNase-free DNase (Promega, Southampton, UK). RNA was reverse transcribed using a Superscript II Preamplification system (Thermo Fisher Scientific). The following primers were used to amplify the target genes (14): IL-8, forward 5’-CGA TGT CAG TGC ATA AAG ACA, reverse 5’-TGA ATT CTC AGC CCT CTT CAA AAA; IL-6, forward 5’-TAC CCC CAG GAG AAG ATT CC, reverse 5’-TTT TCT GCC AGT GCC TCT TT; GAPDH, forward 5’-ACA GTC AGC CGC ATC TTC TT, reverse 5’-GAC AAG CTT CCC GTT CTC AG. cDNA was amplified using a SYBR® GreenER™ qPCR SuperMix Kit (Thermo Fisher Scientific) on the iCycler Thermal Cycler (Bio-Rad Laboratories, Watford, UK).

Supplementary Figures

A

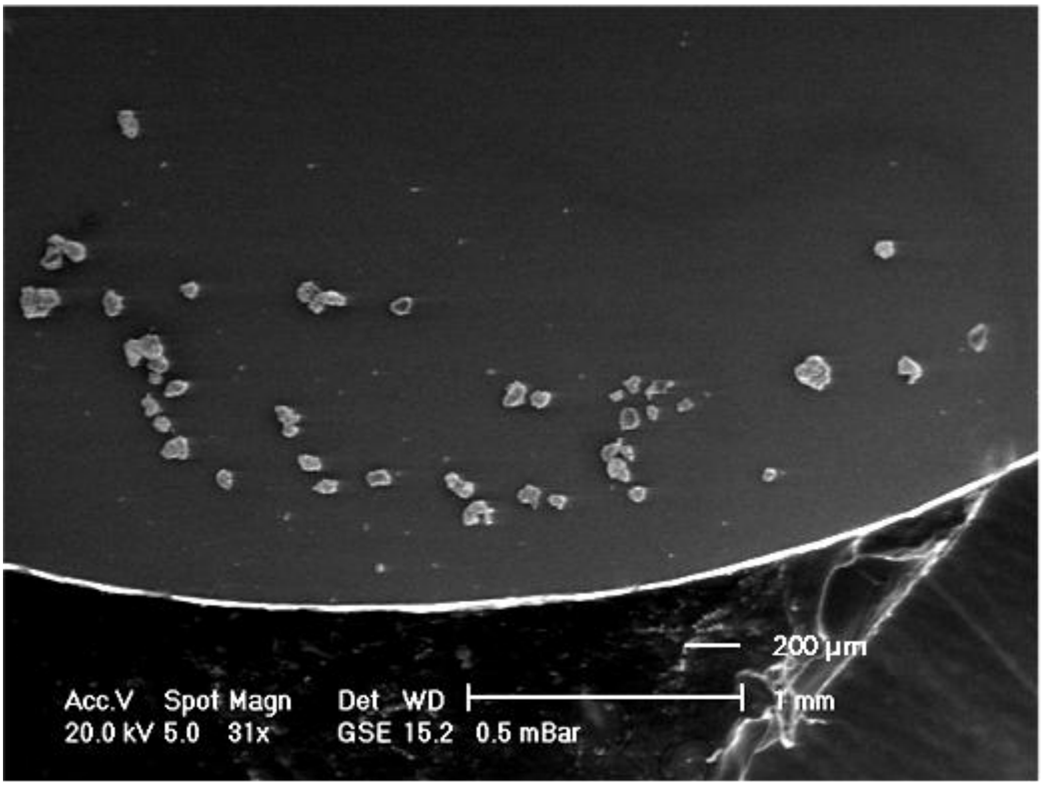

B

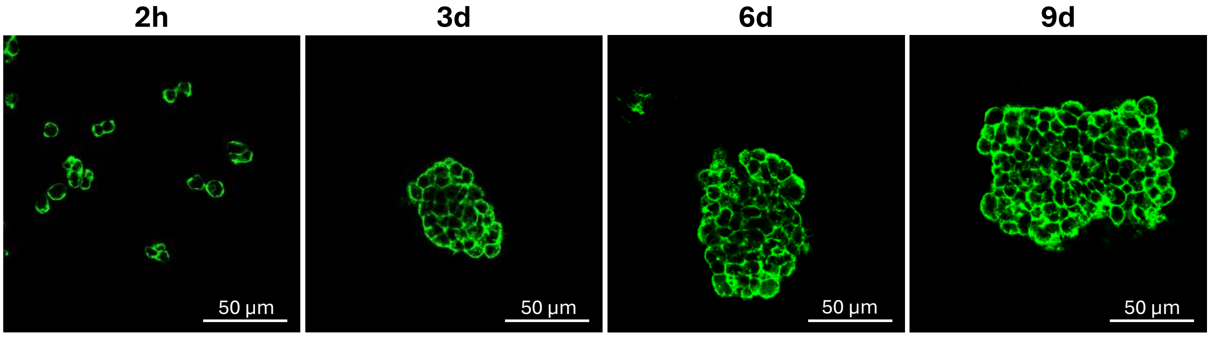

Figure S1. Scanning electron microscopy and fluorescence microscopy of A549 spheroids. (**A**) Scanning electron micrograph of A549 spheroids after 3 days of gyrotatory culture. The image was acquired at 31× magnification using a 20 kV accelerating voltage in low-vacuum mode (0.5 mBar) with a working distance of 15.2 mm. (**B**) Representative confocal micrographs showing F-actin staining in A549 spheroids after 2 hours and 3, 6, and 9 days of culture.
